# Brain Tissue Oxygenation and Cerebral Metabolic Patterns in Focal and Diffuse Traumatic Brain Injury

**DOI:** 10.3389/fneur.2014.00064

**Published:** 2014-05-01

**Authors:** Karlis Purins, Anders Lewén, Lars Hillered, Tim Howells, Per Enblad

**Affiliations:** ^1^Section of Neurosurgery, Department of Neuroscience, Uppsala University, Uppsala, Sweden

**Keywords:** brain tissue oxygenation, cerebral metabolism, traumatic brain injury, cerebral ischemia, Neurovent-PTO

## Abstract

**Introduction:** Neurointensive care of traumatic brain injury (TBI) patients is currently based on intracranial pressure (ICP) and cerebral perfusion pressure (CPP) targeted protocols. There are reasons to believe that knowledge of brain tissue oxygenation (BtipO_2_) would add information with the potential of improving patient outcome. The aim of this study was to examine B_ti_pO_2_ and cerebral metabolism using the Neurovent-PTO probe and cerebral microdialysis (MD) in TBI patients.

**Methods:** Twenty-three severe TBI patients with monitoring of physiological parameters, ICP, CPP, B_ti_pO_2_, and MD for biomarkers of energy metabolism (glucose, lactate, and pyruvate) and cellular distress (glutamate, glycerol) were included. Patients were grouped according to injury type (focal/diffuse) and placement of the Neurovent-PTO probe and MD catheter (injured/non-injured hemisphere).

**Results:** We observed different patterns in B_ti_pO_2_ and MD biomarkers in diffuse and focal injury where placement of the probe also influenced the results (ipsilateral/contralateral). In all groups, despite fairly normal levels of ICP and CPP, increased MD levels of glutamate, glycerol, or the L/P ratio were observed at B_ti_pO_2_ <5 mmHg, indicating increased vulnerability of the brain at this level.

**Conclusion:** Monitoring of B_ti_pO_2_ adds important information in addition to traditional ICP and CPP surveillance. Because of the different metabolic responses to very low B_ti_pO_2_ in the individual patient groups we submit that brain tissue oximetry is a complementary tool rather than an alternative to MD monitoring.

## Introduction

Traumatic brain injury (TBI) remains a major cause of morbidity and mortality (1, 2). The management of TBI patients is largely based on intracranial pressure (ICP) and cerebral perfusion pressure (CPP) targeted treatment protocols in order to prevent secondary brain injury and to improve patient outcome (3–7). Cerebral hypoxia and ischemia frequently occur after severe head injury (8–10) and are major factors causing secondary brain injury (11, 12). The occurrence and duration of both entities are negatively correlated with patient outcome (12–14). Multi-modality monitoring of brain tissue oxygenation (B_ti_pO_2_) and cerebral metabolism [e.g., with microdialysis (MD)], provides information for early detection of brain ischemia and could possibly be used to avoid secondary ischemic brain injury (15). In the last decades, extensive amount of research has been done regarding cerebral MD and Clark-type electrochemical B_ti_pO_2_ monitors (16, 17). Recently, a new fiber optic probe (Neurovent-PTO, NV) was introduced. The device has a great clinical advantage of measuring B_ti_pO_2_, ICP and brain temperature, simultaneously in a single probe. The clinical experience of the new NV probe is not extensively reported and its potential benefits and limitations needs to be further explored. We also need further knowledge to understand the clinical significance of low BtipO_2_ levels. We have previously reported the accuracy and stability of the NV probe *in vitro* (18) and defined the B_ti_pO_2_ threshold level of ischemia (19) in a standardized pig brain death model (20). To our knowledge, however, there is no published study clarifying the relation of B_ti_pO_2_ and cerebral metabolism using the new NV probe and cerebral MD in TBI patients. Therefore, the aim of the present study was to examine the cerebral metabolism and cerebral oxygen levels with MD and the new NV probe. The secondary objective was to determine if the response pattern differed depending on type of injury and probe localization.

## Materials and Methods

### Patient material and neurointensive care

This study included 23 patients (21 men and 2 women) with severe TBI [including cerebral contusions, diffuse axonal injury (DAI), and extracerebral hematomas]. Mean age was 46 years (range 16–82). All patients were admitted to the neurointensive care unit (NICU) at the Uppsala University Hospital between year 2008 and 2012. Patients with Glasgow Coma Scale of ≤8 (not obeying commands or worse) at the NICU were included. CT scans were performed in all patients. All patients received continuous propofol infusion 1–4 mg/kg/h (Propofol-Lipuro^®^, B Braun Melsungen AG, Melsungen, Germany) as sedation and morphine as analgesia, 1–3 mg intermittently (Morfin Meda^®^, Meda, Sollentuna, Sweden). In all patients, advanced multiparameter neuromonitoring was applied for ICP, CPP, B_ti_pO_2_, and for cerebral metabolism. The B_ti_pO_2_ and MD probes were inserted into the brain as soon as possible after the injury or if a patient deteriorated during the stay in NICU (see below). The treatment was based on ICP and CPP guided protocols (ICP <20 mmHg; CPP >60 mmHg) including mild hyperventilation (PaCO_2_ 30–35 mmHg) and head elevation to 30°. Normoventilation was applied as soon as possible. Mass lesions were removed when indicated. High ICP was controlled with hyperventilation, cerebrospinal fluid drainage, barbiturate coma treatment (Pentothal Natrium, Abbott Laboratories, IL, USA), and decompressive craniectomy in an escalated manner.

### Brain tissue oximetry

A multiparameter Neurovent-PTO^®^ (NV) probe (Raumedic, Munchberg, Germany) for continuous measurements of ICP, B_ti_pO_2_, and brain temperature was inserted via a burr hole usually in the right frontal lobe. The probes were placed in the left frontal lobe in cases when hemicraniectomy or evacuation of mass lesion was indicated on that side.

### Microdialysis

**Figure 1:**
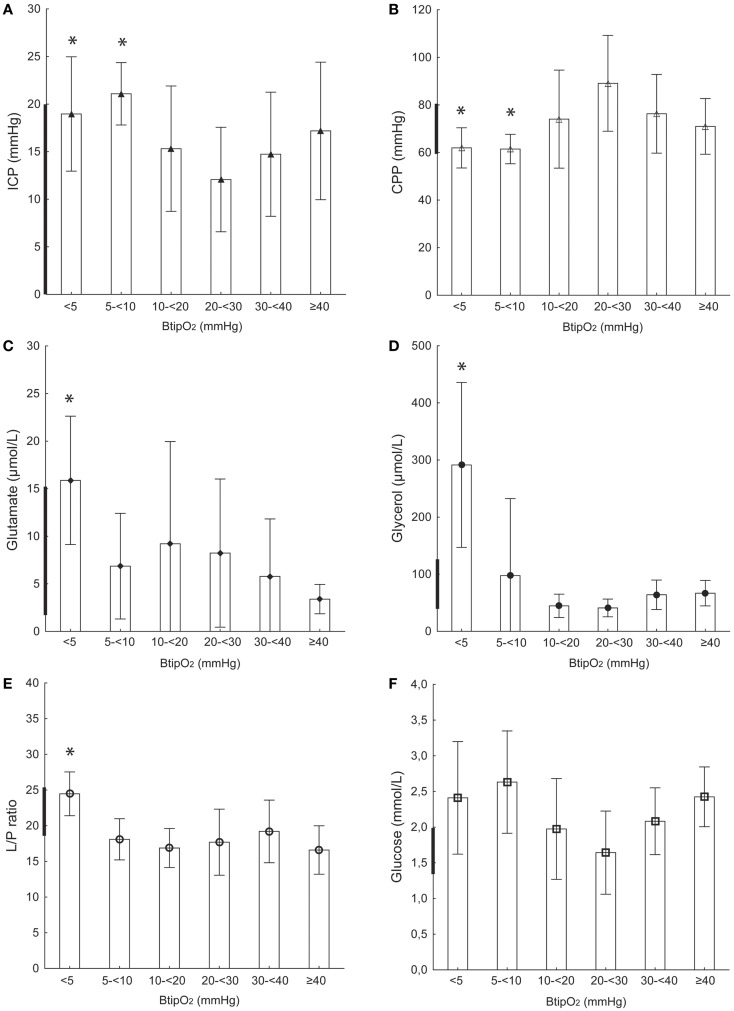
**Focal injury and contralateral probe location**. ICP **(A)**, CPP **(B)**, glutamate **(C)**, glycerol **(D)**, L/P ratio **(E)**, and glucose **(F)** at different B_ti_pO_2_ levels in TBI patients with focal injury and probe (MD and NV) placement on the contralateral side from the injury (non-injured hemisphere). Bold line on *Y*-axis shows tentative normal MD values based on Reinstrup et al. (21) and Schulz et al. (22). All values are expressed as mean ± SD. *Denotes a statistically significant difference (*p* < 0.05) compared to each of the higher B_ti_pO_2_ levels (Mann–Whitney *U* test).

**Figure 2:**
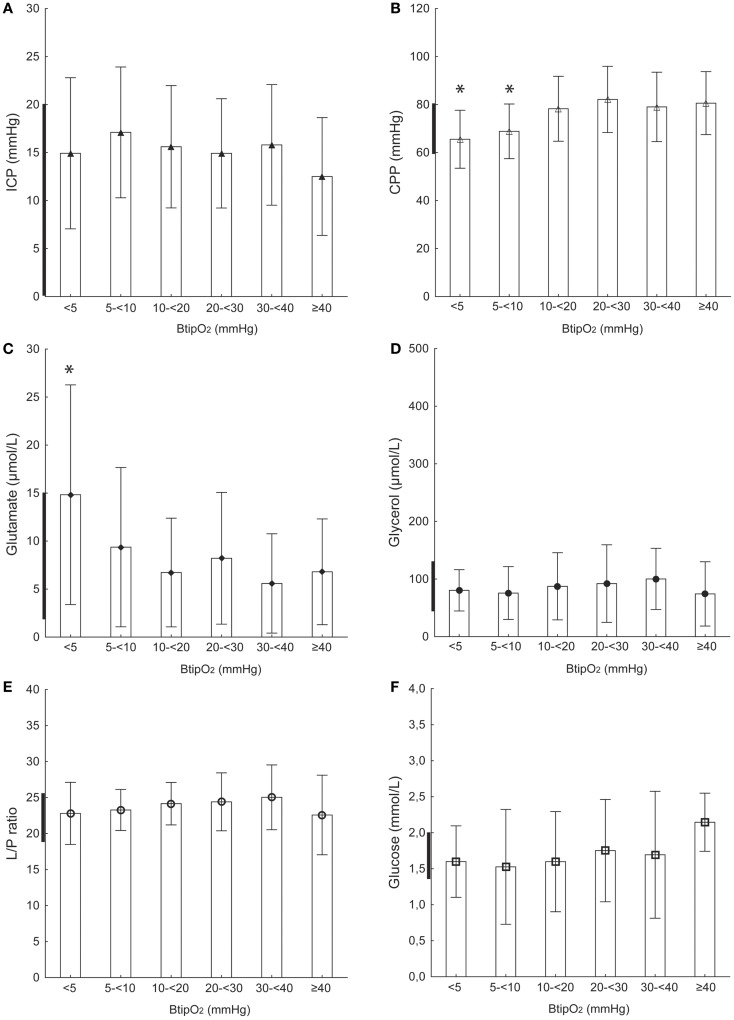
**Focal injury and ipsilateral probe location**. ICP **(A)**, CPP **(B)**, glutamate **(C)**, glycerol **(D)**, L/P ratio **(E)**, and glucose **(F)** at different B_ti_pO_2_ levels in TBI patients with focal injury and probe (MD and NV) placement on the ipsilateral side from the injury (injured hemisphere). Bold line on *Y*-axis shows tentative normal MD values based on Reinstrup et al. (21) and Schulz et al. (22). All values are expressed as mean ± SD. *Denotes a statistically significant difference (*p* < 0.05) compared to each of the higher B_ti_pO_2_ levels (Mann–Whitney *U* test).

**Figure 3:**
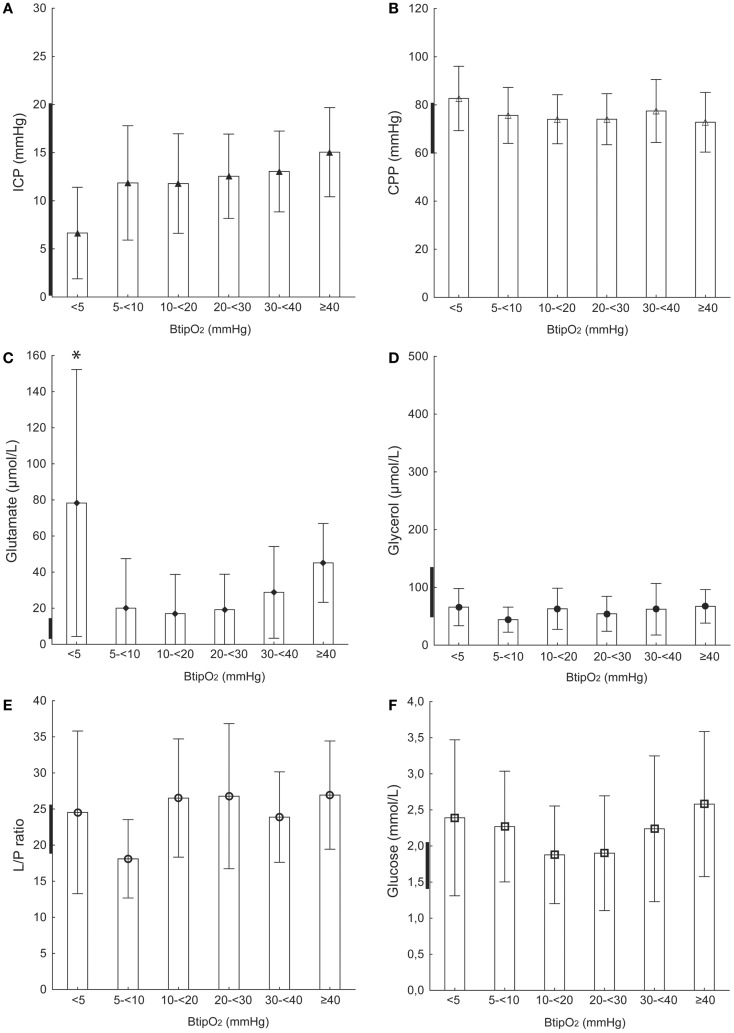
**Diffuse injury and probe location on the right side**. ICP **(A)**, CPP **(B)**, glutamate **(C)**, glycerol **(D)**, L/P ratio **(E)**, and glucose **(F)** at different B_ti_pO_2_ levels. TBI patients with diffuse axonal injury and probe (MD and NV) placements in the right frontal hemisphere. Bold line on *Y*-axis shows tentative normal MD values based on Reinstrup et al. (21) and Schulz et al. (22). All values are expressed as mean ± SD. *Denotes a statistically significant difference (*p* < 0.05) compared to each of the higher B_ti_pO_2_ levels (Mann–Whitney *U* test).

A MD catheter [71 High Cut-Off Brain Microdialysis Catheter, M Dialysis AB (formerly CMA Microdialysis) Solna, Sweden] was placed through a separate burr hole in close proximity to the NV probe. The MD catheter was connected to a microinjection pump (106 MD Pump, M Dialysis AB) and perfused with Perfusion Fluid CNS (M Dialysis AB) with a flow rate of 0.3 μL/min. The MD samples were collected in 1-h intervals and analyzed for lactate, pyruvate, glucose, glutamate, and glycerol with enzymatic techniques using a bedside analyzer (CMA 600, CMA Microdialysis, Solna, Sweden). The analyzers were automatically calibrated when started as well as every sixth hour using standard calibration solutions from the manufacturer. The total imprecision (coefficient of variation) of the analyzed method was <10% for all analytes. In all patients, the first 6 h of the monitoring time was excluded due to the time needed for the measurement stabilization of the B_ti_pO_2_ probe. The time periods with barbiturate induced coma were excluded from this study. Bold line on *Y*-axis (Figures 1–3) shows the tentative normal MD values based on Reinstrup et al. (21) and Schulz et al. (22). No correction for relative recovery (extraction efficiency) was made.

### Classification of type of injury and probe placement

The type of injury was classified as focal (extra cerebral hematomas or contusions) or as DAI (Table 1). The patient group with focal lesions was subdivided into two groups based on probe placement: ipsilateral (injured hemisphere) and contralateral (non-injured hemisphere).

**Table 1:** **Traumatic brain injury patients investigated: type of injury, NV probe, and MD catheter location**.

Patient no.	Extra cerebral hematoma	Cerebral contusions	Diffuse axonal injury	Probe location
1		X		Contra
2		X		Contra
3		X		Contra
4	X			Contra
5	X			Contra
6	X	X		Contra
7	X	X		Ipsi
8	X	X		Ipsi
9	X			Ipsi
10	X			Ipsi
11	X			Ipsi
12	X			Ipsi
13		X		Ipsi
14		X		Ipsi
15		X		Ipsi
16		X		Ipsi
17		X		Ipsi
18		X		Ipsi
19			X	Right side
20			X	Right side
21			X	Right side
22			X	Right side
23			X	Right side

*Ipsi, probe placement frontal in injured hemisphere; contra, probe placement frontal in non-injured hemisphere; right, probe placement in right frontal hemisphere*.

### Data collection and statistical methods

Intracranial pressure, CPP, MD, B_ti_pO_2_, and physiological data (heart rate, arterial blood pressure, and peripheral oxygen saturation) were acquired and processed using the Odin software for multi-modality monitoring in the NICU, developed at Uppsala University and Edinburgh University (23). The trend data were stored in a minute by minute time format. Artifacts, which mainly occurred during the probe recalibration or nursing interventions, were manually removed from the datasets. For the correlative MD data analysis, the continuous data of B_ti_pO_2_ and CPP were averaged for 1-h intervals to match the MD sampling periods taking the 17 min time lag owing to MD catheter dead space into account. Statistical analyses and graphical views were done using Statistica 10.0 for Windows (StatSoft Inc., Tulsa, OK, USA). All data were evaluated for normal distribution and did not meet the assumptions for parametric analysis. Therefore a non-parametric analysis was performed using Kruskal–Wallis analysis of variance (ANOVA) on the full set of evaluated B_ti_pO_2_ levels and MD data, and if this was significant, Mann–Whitney *U* test was used to determine at which levels of B_ti_pO_2_ there were significant differences (ICP, CPP, and MD). Results were considered significant if *p* < 0.05. The data are presented as mean values ± standard deviation (SD).

### Ethics

The regional ethical review board in Uppsala has approved this study for human research. Informed consent to participate in the study was obtained for all patients from the nearest kin.

## Results

The B_ti_pO_2_ and MD probes were inserted 35 ± 23 h (mean ± SD) after the injury. The mean duration of the B_ti_pO_2_ was 199 h (range 13–496 h). Table 2 and Figures 1–3 summarizes the measurements of ICP, CPP, and MD values (L/P ratio, glutamate, glycerol, glucose) at different B_ti_pO_2_ levels for all patient groups. The results are presented divided in focal (with ipsilateral or contralateral measurements) and diffuse injury.

**Table 2:** **Brain tissue oxygenation and MD-dialyzate concentrations at different ICP and CPP levels**.

B_ti_pO_2_	ICP	CPP	Glutamate	Glycerol	L/P ratio	Glucose	Sample size (MD)
**FOCAL INJURY, CONTRALATERAL (n = 6)**							
<5	18.9 ± 6.0*	61.9 ± 8.4*	15.8 ± 6.7*	291.3 ± 144.5*	24.4 ± 3.0*	2.4 ± 0.7	12
5 ≤ 10	21.0 ± 3.2*	61.4 ± 6.1*	6.8 ± 5.5	97.5 ± 134.9	18.0 ± 2.8	2.4 ± 0.7	20
10 ≤ 20	15.3 ± 6.5	74.0 ± 20.6	9.2 ± 10.7	44.6 ± 20.5	16.8 ± 2.7	1.9 ± 0.7	22
20 ≤ 30	12.0 ± 5.4	89.0 ± 20.0	8.2 ± 7.7	41.0 ± 15.5	17.6 ± 4.6	1.6 ± 0.5	9
30 ≤ 40	14.7 ± 6.5	76.2 ± 16.5	5.7 ± 6.0	63.9 ± 25.8	19.2 ± 5.3	2.0 ± 0.4	262
≥40	17.1 ± 7.2	71.0 ± 11.7	3.3 ± 1.5	66.8 ± 22.3	16.5 ± 3.3	2.4 ± 0.4	228
**FOCAL INJURY, IPSILATERAL (n = 12)**							
<5	14.9 ± 7.8	65.5 ± 12.0*	14.8 ± 11.4*	80.4 ± 35.8	22.8 ± 4.3	1.5 ± 0.5	30
5 ≤ 10	17.1 ± 6.8	68.8 ± 11.4*	9.3 ± 8.3	75.7 ± 45.8	23.2 ± 2.8	1.5 ± 0.8	48
10 ≤ 20	15.6 ± 6.3	78.2 ± 13.5	6.7 ± 7.1	87.4 ± 58.3	24.1 ± 2.9	1.6 ± 0.7	152
20 ≤ 30	14.9 ± 5.6	82.1 ± 13.8	8.2 ± 6.8	92.1 ± 67.3	24.4 ± 4.0	1.7 ± 0.7	123
30 ≤ 40	15.8 ± 6.2	79.0 ± 14.4	5.5 ± 5.1	100.1 ± 53.1	25.0 ± 4.5	1.7 ± 0.8	244
≥40	12.5 ± 6.1	80.6 ± 13.3	6.7 ± 5.5	74.2 ± 55.7	22.5 ± 5.5	2.1 ± 0.4	85
**DIFFUSE INJURY (n = 5)**							
<5	6.6 ± 4.7	82.6 ± 13.3	78.2 ± 73.9*	65.8 ± 32.2	24.5 ± 11.2	2.3 ± 1.0	14
5 ≤ 10	11.8 ± 5.9	75.6 ± 11.6	20.0 ± 27.4	44.1 ± 21.7	18.1 ± 5.4	2.2 ± 0.7	33
10 ≤ 20	11.8 ± 5.1	74.0 ± 10.1	17.0 ± 21.7	62.9 ± 35.6	26.5 ± 8.1	1.8 ± 0.6	166
20 ≤ 30	12.5 ± 4.3	74.0 ± 10.6	19.2 ± 19.6	54.3 ± 30.3	26.7 ± 10.0	1.9 ± 0.8	270
30 ≤ 40	13.0 ± 4.2	77.4 ± 25.4	28.7 ± 25.4	62.2 ± 44.5	23.8 ± 6.2	2.2 ± 1.0	129
≥40	15.0 ± 4.6	72.8 ± 12.4	45.1 ± 21.8	67.1 ± 29.0	26.9 ± 7.4	2.5 ± 1.0	45

NV probe and MD catheter placement in focal (ipsi- or contra-lateral side) or diffuse axonal injury (right frontal hemisphere). All values are shown as mean ± SD. *Denotes a statistically significant (*p* < 0.05) difference compared to each of the higher B_ti_pO_2_ levels (Mann–Whitney *U* test).

### Focal brain injury and probe placement on the contralateral side

#### ICP, CPP, and B_ti_pO_2_

During monitoring ICP mean values ranged between 12 and 17 mmHg and CPP was in the range 70–90 mmHg at B_ti_pO_2_ levels of ≥10 mmHg. When B_ti_pO_2_ decreased below 10 mmHg ICP was significantly higher (21.0 ± 3.2 mmHg) and CPP was significantly lower (61.4 ± 6.1 mmHg) (*p* < 0.01) (Table 2; Figures 1A,B).

#### MD-glutamate and B_ti_pO_2_

In this group of patients, MD-glutamate was around 3–9 μmol/L at B_ti_pO_2_ levels higher than 5 mmHg (Table 2; Figure 1C). When B_ti_pO_2_ was below 5 mmHg we observed a significant (*p* < 0.05) increase of MD-glutamate (15.8 ± 6.7 μmol/L).

#### MD-glycerol and B_ti_pO_2_

Similarly, in all patients in this group MD-glycerol was 44–66 μmol/L when B_ti_pO_2_ was >10 mmHg. B_ti_pO_2_ 5–10 mmHg was accompanied with a slight increase of MD-glycerol to 97.5 ± 134.9 μmol/L (Table 2; Figure 1D). A significant increase of MD-glycerol to 291.3 ± 144.5 (*p* < 0.01) was observed at very low oxygen levels ( <5 mmHg).

#### MD–L/P ratio and B_ti_pO_2_

The MD–L/P ratio remained stable and within the normal levels (mean range 16–19) when B_ti_pO_2_ was >5 mmHg. The MD–L/P ratio was significantly increased to 24.4 ± 3.0 (*p* < 0.01) at B_ti_pO_2_ levels below 5 mmHg (Table 2; Figure 1E).

#### MD-glucose and B_ti_pO_2_

Table 2 and Figure 1F shows the mean MD-glucose concentrations at different B_ti_pO_2_ levels. We did not observe any significant differences between MD-glucose and B_ti_pO_2_.

### Focal brain injury and probe placement on the ipsilateral side

#### ICP, CPP, and B_ti_pO_2_

Table 2 and Figure 2A show the ICP corresponding to different B_ti_pO_2_ levels in patients with a focal injury and probe placement in the ipsilateral side. Mean ICP was within the normal levels, between 12 and 17 mmHg for all levels of B_ti_pO_2_. The lowest mean ICP (12.5 ± 6.1 mmHg) was seen at the highest B_ti_pO_2_ levels ( ≥40 mmHg), but the differences were not statistically significant.

The mean CPP values with B_ti_pO_2_ levels below 10 mmHg were significantly *p* < 0.05) lower than mean CPP at B_ti_pO_2_ >10 mmHg (Table 2; Figure 2B). When the B_ti_pO_2_ levels were between 5 and 10 mmHg, the mean CPP was 68.8 ± 11.4 mmHg. And at the B_ti_pO_2_ level below 5 mmHg the mean CPP was 65.5 ± 12.0 mmHg. At B_ti_pO_2_ levels above 10 mmHg the CPP levels were stable and somewhat high (mean range 78–82 mmHg).

#### MD-glutamate and B_ti_pO_2_

The mean MD-glutamate concentration ranged between 5 and 8 μmol/L at B_ti_pO_2_ above 10 mmHg. A slight but not significant increase in MD-glutamate concentration was observed at levels of B_ti_pO_2_ 5–10 mmHg (Table 2; Figure 2C). A drop of B_ti_pO_2_ below 5 mmHg resulted in a significant increase (*p* < 0.05) of MD-glutamate concentration (14.8 ± 11.4 μmol/L).

#### MD-glycerol and MD–L/P ratio

No differences were seen between B_ti_pO_2_ levels MD-glycerol. No significant differences observed between B_ti_pO_2_ and MD–L/P ratio (Table 2; Figures 2D,E).

#### Mean MD-glucose

Mean MD-glucose levels were 2.1 ± 0.4 mmol/L at the highest B_ti_pO_2_ pressure and had a stepwise decreasing trend to 1.5 ± 0.5 mmol/L at B_ti_pO_2_ levels 0–5 mmHg (Table 2; Figure 2F). This difference did not reach statistical significance.

### Diffuse axonal injury

#### ICP, CPP, and B_ti_pO_2_

In patients with DAI, mean ICP ranged from 11 to 15 mmHg when B_ti_pO_2_ levels were >5 mmHg (Table 2; Figure 3A). At the level of B_ti_pO_2_ below 5 mmHg the mean ICP was even lower (6.6 ± 4.7 mmHg) but not statistically significant. At all B_ti_pO_2_ levels CPP remained within normal levels ranging from 72 to 82 mmHg (Table 2; Figure 3B). No correlation was found between CPP and B_ti_pO_2_ levels.

#### MD-glutamate and B_ti_pO_2_

MD-glutamate was abnormally high in all patients within this group (Figure 3C). It was significantly higher (78.2 ± 73.9 μmol/L; *p* < 0.05) when B_ti_pO_2_ level was <5 mmHg (compared to B_ti_pO_2_ ≥5 mmHg) (Table 2; Figure 3C). At B_ti_pO_2_ levels of ≥5 mmHg the mean MD-glutamate concentrations had no significant correlation with B_ti_pO_2_ and varied between 17.0 and 45.1 μmol/L.

#### MD-glycerol and B_ti_pO_2_

Mean MD-glycerol concentration ranged between 44.1 and 67.1 μmol/L and had no significant correlation between different B_ti_pO_2_ levels (Table 2; Figure 3D).

#### MD–L/P ratio and B_ti_pO_2_

Table 2 and Figure 3E presents the MD–L/P ratio correlation to different B_ti_pO_2_ levels. The mean MD–L/P ratio values ranged between 18.1 and 26.9 and had no relation to B_ti_pO_2_.

#### MD-glucose and B_ti_pO_2_

Table 2 and Figure 3F shows the MD-glucose concentrations in relation to different B_ti_pO_2_ levels. MD-glucose remained normal at all B_ti_pO_2_ levels and varied between 1.8 and 2.5 mmol. Differences in MD-glucose levels did not reach statistical significance.

## Discussion

We found that there were different response patterns of B_ti_pO_2_ and cerebral metabolism depending on the injury type and probe localization which will be discussed in the following sections. Monitoring of B_ti_pO_2_ in TBI patients is of considerable clinical interest, but the exact threshold level of ischemia has been difficult to establish despite progress on methodological issues. Measurements of B_ti_pO_2_ using the recently introduced NV probes have shown to be safe and reliable in several experiments (18–20, 24). In a pre-clinical study, we found that impaired cerebral metabolism determined according to intracerebral MD levels occurred at a B_ti_pO_2_ level below 10 mmHg using the new NV probe (19). However, to our knowledge, there are no previously published clinical studies using NV probes together with MD in order to relate cerebral metabolism with critical B_ti_pO_2_ levels in TBI patients. Therefore, the objective of this investigation was to study B_ti_pO_2_ and cerebral metabolism at different levels of ICP and CPP in patients with focal and diffuse TBI using the new NV probe.

### Brain tissue oxygenation and intracranial dynamics

Results from our previous pre-clinical study showed a simultaneous decrease of CPP and B_ti_pO_2_ during a gradual increase of the ICP (19). There was also a threshold level of impaired cerebral metabolism observed at B_ti_pO_2_ <10 mmHg. In the present study, we found a significant correlation between low B_ti_pO_2_ values ( <10 mmHg) and increased ICP (mean range 19–21 mmHg) or decreased CPP (mean 61 mmHg) in TBI patients with focal injury and the probe placed in the un-injured hemisphere (Figure 1; Table 2). In patients with focal injury and probe placement in the ipsilateral hemisphere, periods with B_ti_pO_2_ <10 mmHg showed significantly lower CPP but there was no relation to ICP (Figure 2; Table 2). In patients with diffuse injury periods with B_ti_pO_2_ <5 mmHg tended to be associated with lower ICP but there was no relation to CPP. Thus, the type of injury and probe placement appear to be factors determining the relation between B_ti_pO_2_ and ICP and CPP, respectively.

### L/P ratio

The L/P ratio is a balance between lactate and pyruvate reflecting the state of cerebral oxidative metabolism and is known as a sensitive marker of cerebral ischemia (25–28). MD–L/P ratio was recently reported to be an independent positive predictor of poor outcome in a large cohort of TBI patients (29). Normal MD–L/P ratio values have been reported previously as approximately 15–20 (21, 22, 30, 31). Prior studies have used different L/P ratio threshold levels of cerebral ischemia ranging from 25 to 40 (17, 19, 32, 33). Results from a study of focal TBI revealed that the L/P ratio values are higher in the tissue “at-risk” (ipsilateral side) compared to “normal” tissue (contralateral side) (32). Similarly, in the current study L/P ratio seemed to be higher at all B_ti_pO_2_ levels in TBI patients with focal injury and when probes were placed in the ipsilateral side and also in DAI patients. However, a decrease of B_ti_pO_2_ to very low levels ( <5 mmHg) resulted in a significant increase of L/P ratio only in patients with focal brain injury and when probes were placed in contralateral side but not in the ipsilateral side (Figures 1E and 2E). Thus, a somewhat provocative assumption appeared that in the case of injured tissue in which the L/P ratio is already elevated, very low B_ti_pO_2_ levels do not lead to even higher L/P ratios. In relatively un-injured tissue (contralateral side) with a normal L/P ratio, however, very low B_ti_pO_2_ levels do lead to increases in the L/P ratio. Further studies with increased number of patients are needed to support this hypothesis.

### Glutamate

Glutamate is the main excitatory transmitter of the central nervous system. In a healthy brain it is actively taken up by neurons and astrocytes after it is released in the synaptic cleft. A significant increase in interstitial glutamate indicates impaired cerebral energy metabolism and impending cell damage (34). Increased brain interstitial MD-glutamate has been reported earlier in TBI (17, 35–40).

MD-glutamate basal concentration in humans ranges between 5 and 15 μmol/L (21, 22). A clinical B_ti_pO_2_ TBI study identified MD-glutamate as the most sensitive and early marker of cerebral ischemia (15). In that study, a significant increase of MD-glutamate was observed at B_ti_pO_2_ level below 10 mmHg. A positive correlation has been demonstrated in TBI patients between high levels of MD-glutamate and increased ICP and poor outcome (41). Increased MD-glutamate levels have also been reported in TBI patients mostly with CPP below 70 mmHg (37). In that study, the authors also found patients with high MD-glutamate and CPP above 70 mmHg. In addition, they did not specify the location of the probe and the type of the injury which could explain potentially different pathophysiological processes. The effect of MD catheter location has also been studied in TBI patients with focal injury, and higher MD-glutamate concentrations were found in the most injured brain hemisphere (42).

In the present study in patients with focal TBI, we observed that MD-glutamate increased significantly irrespective of the placement of the probe when B_ti_pO_2_ decreased to extremely low ( <5 mmHg) levels. DAI patients appeared to have higher MD-glutamate levels than patients with a focal injury, but even in this group a decrease of B_ti_pO_2_ to <5 mmHg was associated with significantly higher glutamate levels (Figure 3C), illustrating the energy dependence of the astrocytic glutamate-glutamine cycle capacity to clear interstitial glutamate (34). The overall high MD-glutamate concentration observed in diffuse injury could be explained by massive neuronal cell damage occurring in DAI and most likely originating from intracellular stores that leak into the extra-cellular space as the neuronal membrane loses its structural integrity (43).

### Glycerol

Glycerol is one of the end products in cell membrane phospholipid degradation. It can be used as a marker of phospholipid degradation in cerebral ischemia (44). Experimental and clinical studies have shown significant increases of MD-glycerol during cerebral ischemia (44–46). Normal cerebral MD-glycerol levels have been reported previously from patients during wakefulness, anesthesia, and neurosurgical procedures (21). Clausen et al. reported increased glycerol levels when B_ti_pO_2_ decreased below 10 mmHg (47). We have recently shown in a pre-clinical study that the interstitial MD-glycerol concentration increases when CPP or B_ti_pO_2_ decrease (19). In the present study under similar conditions (focal TBI on the contralateral side), periods with low CPP and B_ti_pO_2_ significantly correlated with increased MD-glycerol levels. However, we did not see any correlation of B_ti_pO_2_ and MD-glycerol levels in the more injured hemisphere (ipsi) or in DAI patients. These results are similar to those for L/P ratio in that relatively un-injured tissue seemed to be more sensitive to decreased B_ti_pO_2_ than injured tissue. It is unclear which factors are responsible for the heterogeneity of the MD-glycerol levels between hemispheres in focal TBI and between focal TBI and DAI patients. Based on recent validation data implicating MD-glycerol as a biomarker of oxidative stress we submit that this may be an important additional factor to consider (48).

### Clinical aspects

In a clinical situation B_ti_pO_2_ may be influenced by parameters such as cerebral metabolism, cerebral blood flow, oxygen diffusion, sedation, hyperventilation, low inspired oxygen, ICP and CPP changes, age, trauma severity, and other traumatic changes in the cellular environment. It is obvious that the interpretation of B_ti_pO_2_ needs to be carefully considered, preferably together with other parameters such as MD and cerebral blood flow assessments. The thresholds may vary in different patients. However, in all three patient groups analyzed very low B_ti_pO_2_ levels <5 mmHg were accompanied by increases in either the MD–L/P ratio, MD-glutamate or MD-glycerol, indicating an increased vulnerability of the brain at this level of oxygen despite fairly normal levels of ICP and CPP. Thus, B_ti_pO_2_ monitoring adds valuable information about the brain vulnerability not disclosed by routine ICP and CPP surveillance. However, very low B_ti_pO_2_ levels <5 mmHg were associated with different response patterns for biomarkers of energy metabolism (MD-glucose, MD–L/P ratio) and cellular distress (MD-glutamate, MD-glycerol) suggesting that B_ti_pO_2_ monitoring is a complement to MD monitoring rather than an alternative.

### Limitations of the study and future direction of research

A number of limitations of this study should be recognized. First, the limited number of patients did not permit multivariate statistical analysis comparing other factors such as carbon dioxide partial pressure (pCO_2_), sedation, and inspired oxygen concentration. Second, we could not address the dependence of oxygen metabolism, oxygen extraction fraction, and cerebral blood flow including auto-regulation. Third, brain temperature data were not analyzed in this paper for technical reasons. Future TBI studies are needed to explore how episodes of low or high B_ti_pO_2_ levels are correlated with hyperventilation, sedation, changes in inspired oxygen concentration, blood pressure, cerebral auto-regulation, cerebral blood flow, and oxygen metabolism as well as outcome. To gather a sufficient number of patients a multi-center study may be needed.

## Conclusion

We observed different patterns of changes in B_ti_pO_2_ and cerebral MD biomarker patterns in focal and diffuse TBI patients. The placement of the probe in focal injury did also influence the results. However, despite fairly normal levels of ICP and CPP in all groups, increased cerebral MD levels of glutamate, glycerol, or the lactate/pyruvate ratio were observed at B_ti_pO_2_ <5 mmHg, indicating increased vulnerability of the brain at this critical level of tissue oxygenation.

## Conflict of Interest Statement

The authors declare that the research was conducted in the absence of any commercial or financial relationships that could be construed as a potential conflict of interest.
